# The Selective Angiotensin II Type 2 Receptor Agonist, Compound 21, Attenuates the Progression of Lung Fibrosis and Pulmonary Hypertension in an Experimental Model of Bleomycin-Induced Lung Injury

**DOI:** 10.3389/fphys.2018.00180

**Published:** 2018-03-27

**Authors:** Anandharajan Rathinasabapathy, Alana Horowitz, Kelsey Horton, Ashok Kumar, Santhi Gladson, Thomas Unger, Diana Martinez, Gaurav Bedse, James West, Mohan K. Raizada, Ulrike M. Steckelings, Colin Sumners, Michael J. Katovich, Vinayak Shenoy

**Affiliations:** ^1^Department of Pharmacodynamics, University of Florida, Gainesville, FL, United States; ^2^Allergy, Pulmonary and Critical Care Medicine, Vanderbilt University Medical Center, Nashville, TN, United States; ^3^Anatomy, University of California at San Francisco, San Francisco, CA, United States; ^4^Department of Physiology and Functional Genomics, University of Florida, Gainesville, FL, United States; ^5^Cardiopulmonary Vascular Biology Lab, Providence VA Medical Center, Brown University, Providence, RI, United States; ^6^Cardiovascular Research Institute Maastricht School for Cardiovascular Diseases, Maastricht University, Maastricht, Netherlands; ^7^Psychiatry and Behavioral Sciences, Vanderbilt University Medical Center, Nashville, TN, United States; ^8^Department of Cardiovascular and Renal Research, Institute for Molecular Medicine, University of Southern Denmark, Odense, Denmark; ^9^Department of Pharmaceutical and Biomedical Sciences, California Health Sciences University, Clovis, CA, United States

**Keywords:** pulmonary fibrosis, pulmonary hypertension, C21, AT_2_ receptor, bleomycin, rats

## Abstract

Idiopathic Pulmonary Fibrosis (IPF) is a chronic lung disease characterized by scar formation and respiratory insufficiency, which progressively leads to death. Pulmonary hypertension (PH) is a common complication of IPF that negatively impacts clinical outcomes, and has been classified as Group III PH. Despite scientific advances, the dismal prognosis of IPF and associated PH remains unchanged, necessitating the search for novel therapeutic strategies. Accumulating evidence suggests that stimulation of the angiotensin II type 2 (AT_2_) receptor confers protection against a host of diseases. In this study, we investigated the therapeutic potential of Compound 21 (C21), a selective AT_2_ receptor agonist in the bleomycin model of lung injury. A single intra-tracheal administration of bleomycin (2.5 mg/kg) to 8-week old male Sprague Dawley rats resulted in lung fibrosis and PH. Two experimental protocols were followed: C21 was administered (0.03 mg/kg/day, ip) either immediately (prevention protocol, BCP) or after 3 days (treatment protocol, BCT) of bleomycin-instillation. Echocardiography, hemodynamic, and Fulton's index assessments were performed after 2 weeks of bleomycin-instillation. Lung tissue was processed for gene expression, hydroxyproline content (a marker of collagen deposition), and histological analysis. C21 treatment prevented as well as attenuated the progression of lung fibrosis, and accompanying PH. The beneficial effects of C21 were associated with decreased infiltration of macrophages in the lungs, reduced lung inflammation and diminished pulmonary collagen accumulation. Further, C21 treatment also improved pulmonary pressure, reduced muscularization of the pulmonary vessels and normalized cardiac function in both the experimental protocols. However, there were no major differences in any of the outcomes measured from the two experimental protocols. Collectively, our findings indicate that stimulation of the AT_2_ receptor by C21 attenuates bleomycin-induced lung injury and associated cardiopulmonary pathology, which needs to be further explored as a promising approach for the clinical treatment of IPF and Group III PH.

## Introduction

Idiopathic Pulmonary Fibrosis (IPF) is a chronic, progressive interstitial lung disease with a median survival of 3 years after diagnosis (Raghu et al., 2011). The origin of IPF remains unknown; however, smoking, viral infections, pollutants, and aging have been identified as potential risk factors (Wolters et al., 2014). Pulmonary hypertension (PH) is a common comorbidity in patients with IPF where it is classified as Group III PH by the World Health Organization (WHO). In fact, PH associated with IPF is considered as one of the principal predictors of mortality (Collum et al., 2017). The prevalence of IPF is growing at an alarming rate worldwide (Ley and Collard, 2013) and the available medical options are very limited. Currently, nintedanib (inhibits multiple tyrosine kinase) and pirfenidone (downregulates transforming growth factor β) are the only drugs approved for IPF treatment (Raghu and Selman, 2015). However, they provide little therapeutic efficacy, fail to prolong survival, and are associated with increased incidence of side effects (Canestaro et al., 2016). All these factors necessitate the search for novel strategies to effectively combat IPF and associated cardiopulmonary symptoms.

The renin angiotensin system (RAS) is a major driving force in maintaining renal, metabolic, and cardiovascular homeostasis. The members of the RAS family have been recently characterized into two major arms that exert opposing actions to one another (Iwai and Horiuchi, 2009)—(a) the deleterious ACE/Ang II/AT_1_ receptor axis, which comprises of Angiotensin converting enzyme (ACE), Angiotensin II (Ang II), and the Angiotensin type 1 (AT_1_) receptor; and, (b) the protective ACE2/Ang-(1-7)/Mas axis, consisting of Angiotensin converting enzyme 2 (ACE2), Angiotensin-(1-7) [Ang-(1-7)], and the Mas receptor. Interestingly, the beneficial effects of activating the ACE2/Ang-(1-7)/Mas axis have been associated with a concomitant increase in the expression of Angiotensin type 2 (AT_2_) receptor (Shenoy et al., 2014), suggesting an active role for this receptor against tissue/organ damage.

Consistent with the aforementioned observations, stimulation of the AT_2_ receptor by a selective ligand, Compound 21 (C21) was shown to confer protection against experimental models of kidney damage (Patel et al., 2016), myocardial infarction (Kaschina et al., 2008), ischemic stroke (Joseph et al., 2014), and islet cell injury (Wang L. et al., 2017). C21 is a highly selective angiotensin II AT_2_ receptor agonist with a K_i_ value of 0.4 nM for the AT_2_ receptor and a K_i_ > 10 mM for the AT_1_ receptor (Wan et al., 2004). We have recently reported that treatment with C21 (0.03 mg/kg/day, ip) after the establishment of disease pathogenesis effectively arrests the progression of monocrotaline-induced PH, and these beneficial effects are abolished by co-administration of the AT_2_ receptor antagonist, PD123319 (Bruce et al., 2015). Though the effects of C21 have been evaluated against a wide variety of disease conditions and organ fibrosis (Wang Y. et al., 2017), its actions against PF and Group III PH are yet to be investigated. Hence, in this study, we investigated the therapeutic potential of C21 against lung fibrosis and associated cardiopulmonary complications in the bleomycin-model of lung injury. The bleomycin animal model mimics key pathophysiological features of human IPF and associated PH following intra-tracheal challenge (Nogueira-Ferreira et al., 2016).

## Materials and methods

### Reagents and chemicals

Bleomycin sulfate (BLEO) was purchased from EMD Millipore (Billerica, MA). C21 was a kind gift from Vicore Pharma (Gothenburg, Sweden). α-smooth muscle actin (α-SMA) and CD68 antibodies were purchased from Abcam (Cambridge, MA, USA) and Dako (Santa Clara, CA, USA), respectively.

### Animals

All animal procedures were approved by the Institutional Animal Care and Use Committee at the University of Florida, complied with National Institutes of Health guidelines and were performed in accordance with the Guide for the Care and Use of Laboratory Animals (Eight Edition, 2011, published by National Academics Press, 500 Fifth Street NW, Washington, DC 20001, USA).

### Study design

Two different protocols were followed in this study—Prevention and Treatment. For both the protocols, 8-week old male Sprague Dawley rats were used (Charles River Laboratories, Wilmington, MA). A pilot experiment was initially performed to determine the best time to initiate C21 injection for the treatment protocol. In this pilot study, PF and accompanying PH (Group III) was induced by a single intra-tracheal injection of BLEO (2.5 mg/kg ≡ 4U/kg, *n* = 4), while control animals received saline (*n* = 4). Three days after BLEO instillation, right ventricular (RV) hemodynamics was measured, and lung tissues were processed for histological examination. Based on this pilot study, it was decided to initiate C21 treatment (0.03 mg/kg/day, ip) after 3 days of bleomycin-instillation (treatment protocol, BCT). However, for the prevention protocol C21 was administered immediately after bleomycin injection (prevention protocol, BCP). For both the protocols, ECHO assessment of RV function and hemodynamics were performed after 2 weeks of BLEO instillation. Lung and heart tissues were harvested for RNA and histological analysis. A subsequent experiment (both prevention and treatment) was performed to obtain all the five lung lobes for estimation of total lung collagen deposition. We have previously demonstrated that C21 was therapeutically active in the monocrotaline model of PH at a dose, 0.03 mg/kg/day (Bruce et al., 2015). At this dose, C21 did not alter any of the following parameters - basal systemic blood pressure, basal right ventricular systolic pressure, cardiac function, and gene expression in normal animals. Both C21 and BLEO were dissolved in sterile deionized water. The terminology AT_2_ (human) has been used throughout the article to denote angiotensin type 2 receptor, except for gene expression studies, wherein, Agtr2 (rat) has been used.

### Echocardiography and hemodynamic assessments

Transthoracic echocardiography was performed after 2 weeks of BLEO instillation to assess ventricular dimensions and cardiac function using a GE Vivid7 ultrasound machine with a 12-MHz transducer (GE Healthcare, NJ, USA). Following echocardiography, the right ventricular systolic pressure (RVSP) was measured as described previously (Rathinasabapathy et al., 2016). Subsequently, animals were sacrificed and organs were harvested for RNA, histology and hypertrophy assessments. Right ventricular hypertrophy [RVH = RV/(LV+S)] was calculated as the ratio of wet weight of right ventricle (RV) and left ventricle + intra-ventricular septum (LV+S).

### Real-time quantitative RT-PCR analysis

Real time qRT-PCR was performed using Taqman Gene Expression System (Life Technologies, USA) to evaluate gene expression of cytokines, fibrotic and extracellular matrix (ECM) markers *viz*. collagen type 1 (Col1a1), collagen type 3 (Col3a1), connective tissue growth factor (Ctgf), interleukin 13 (Il-13), matrix metalloproteinases 12 (Mmp12), tissue inhibitor of metalloproteinases 1 (Timp1), monocyte chemotactic protein 1 (Ccl2), interleukin 6 (Il-6), toll like receptor 4 (Tlr4), and angiotensin receptor type 2 (Agtr2). Total RNA and cDNA preparation was performed as described previously (Anandharajan et al., 2009). Gene expression was calculated by the ΔΔCT method and data was presented as relative fold change from that of control animals. The rat primers have been listed in Table 1.

**Table 1:** Rat primers used for RT-PCR experiment.

**No**	**Gene**	**Sequence**
1.	Col1a1	Rn01463848_m1
2.	Col3a1	Rn01437681_m1
3.	Ctgf	Rn01537279_g1
4.	Mmp12	Rn00588640_m1
5.	Timp1	Rn01430873_g1
6.	IL-13	Rn00587615_m1
7.	Ccl2	Rn00580555_m1
8.	IL-6	Rn01410330_m1
9.	Tlr4	Rn00569848_m1
10.	Agtr2	Rn00560677_s1
11.	18S	Hs99999901_s1

### Histochemical analysis

Following hemodynamic measurements, the left lobe of the lung was inflated with phosphate-buffered saline (PBS), followed by 10% neutral buffered formalin and stored in formalin overnight. Subsequently, the fixed lung tissue was processed (4 μm section) and stained with picro-sirius red. Images were photographed at 10X magnification using an Aperio Imagescope (Leica Biosystems, US). Using Image-J software, the percent of fibrotic area was assessed. To determine remodeling of the lung tissue, sectioned lung specimens (4 μm) were stained with hematoxylin-eosin (H&E) and imaged. Blinded Ashcroft scoring system was performed to grade the tissue remodeling across all experimental groups (Ashcroft et al., 1988). A minimum of 10 non-overlapping images was randomly chosen from each lung section for picro-sirius and H&E staining. For macrophage and muscularization analysis, lung sections (5 μm) were stained with CD68 (1:100) and α-smooth muscle actin (1:200) antibody, respectively, and imaged at 10X (15 random fields/lung section). The degree of muscularization was determined by quantifying the amount of α-smooth muscle actin in different sized vessels (vessel inner diameter—0–25, 25–50, and 50–100 μm). Briefly, when more than 70% of the pulmonary vessel wall is stained by smooth muscle actin, it is classified as completely muscularized, while if it is <70%, it is classified as partially muscularized. Results from each animal was averaged for the subsequent statistical analysis.

### Collagen estimation—hydroxyproline assay

Hydroxyproline assay was performed to estimate lung collagen deposition, as an index of fibrosis. In a separate set of experiments, all the five lung lobes were harvested from each animal, and dried at 65°C for 3 h. Subsequently, the dried lungs were weighed and subjected to collagen estimation according to the protocol provided in the assay kit (Biovisions, CA).

### Statistics

**Figure 1:**
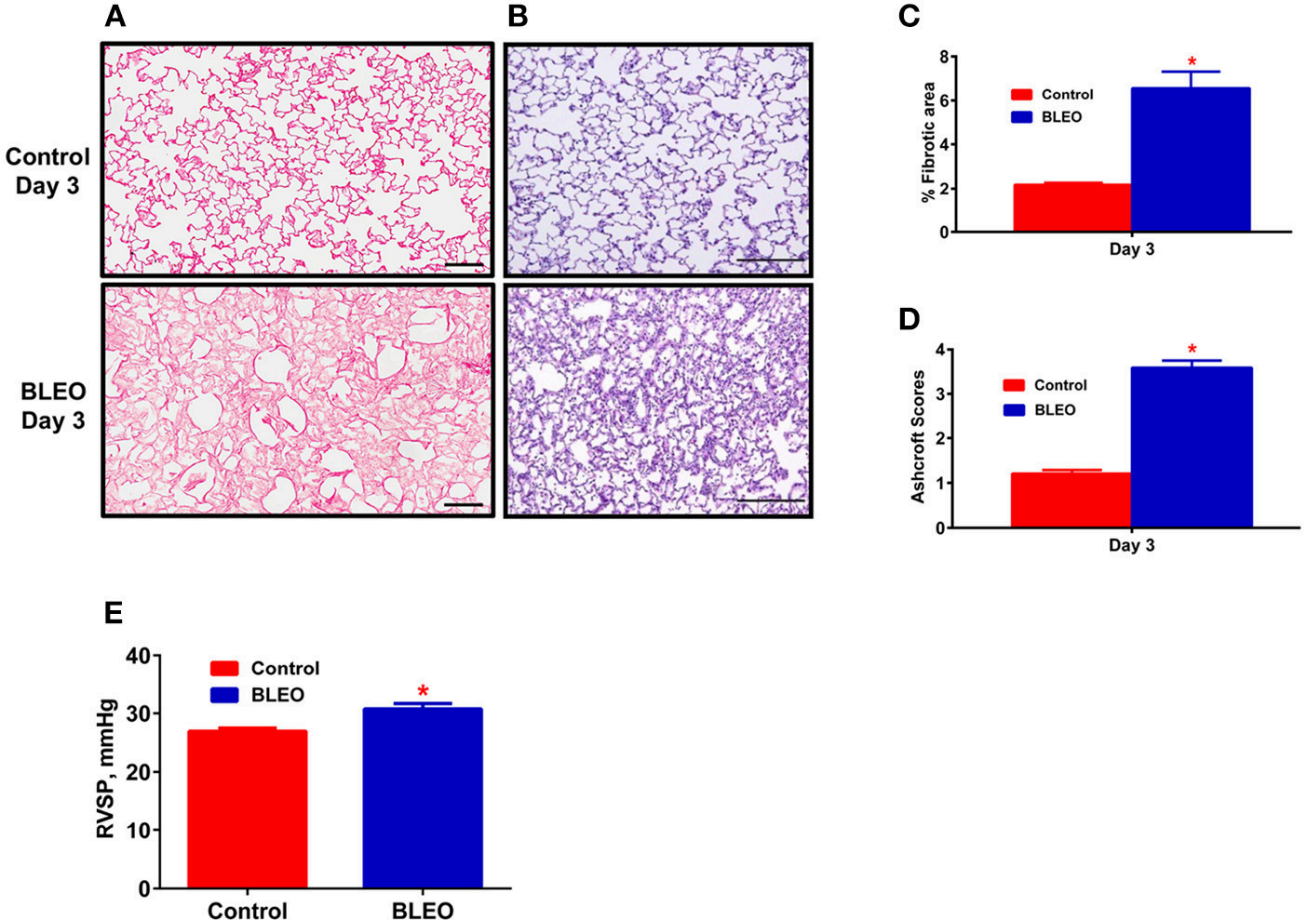
Early onset of lung tissue remodeling and pulmonary hypertension in BLEO animals. **(A,B)** Representative images demonstrating picro-sirius and hematoxylin-eosin staining. **(C,D)** Summary data showing the lung tissue remodeling and fibrosis. **(E)** Summary data showing RVSP after 3 days of BLEO instillation. The data presented in **(C–E)** are mean ± SEM (*n* = 4). ^*^Represents a *p*-value of ≤ 0.05 when comparing BLEO animals against the controls. **(A)** Scale bar = 100 μm and **(B)** Scale bar = 200 μm.

Graph Pad Prism, version 5.0 (La Jolla, CA) was used for statistical analysis. A simple student *t*-test was performed to analyze the data presented in Figure 1. One-way ANOVA followed by Newman-Keuls *post-hoc* analysis test was carried out for all the other end point experimental parameters. Values are represented as means ± SEM, *p***-**values ≤ 0.05 were considered statistically significant.

## Results

### C21 treatment attenuates lung fibrosis and tissue remodeling

**Figure 2:**
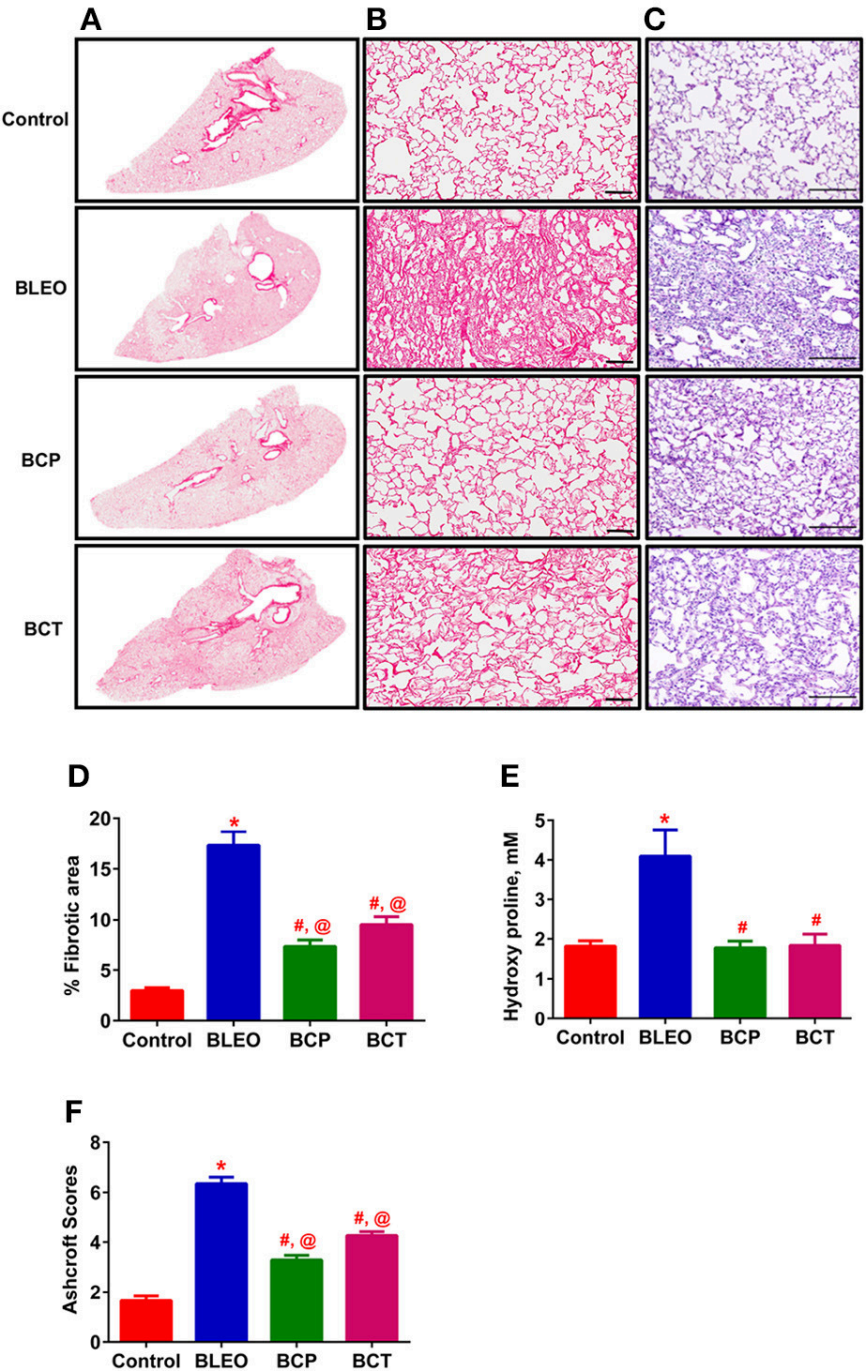
C21 treatment attenuates lung fibrosis and tissue remodeling in BLEO animals. **(A)** Picro-sirius stained images of the whole lung; Con, BLEO, BCP, and BCT. **(B)** Enlarged sections of picro-sirius stained lungs. Scale bar = 100 μm. **(C)** Representative H&E images demonstrating the lung tissue remodeling. Scale bar = 200 μm. **(D,F)** Summary data showing the improvement in lung fibrosis and tissue remodeling in BLEO animals in presence of C21. **(E)** Summary data showing the collagen synthesis estimated by measuring hydroxyproline. The data presented in **(D–F)** are mean ± SEM, from 5, 4–6, and 7–8 animals, respectively. ^*^Indicates a *p*-value of ≤ 0.05, comparing BLEO animals against the controls. #Indicates a *p*-value of ≤ 0.05, comparing BCP and BCT vs. BLEO. @ Indicates a *p*-value of ≤ 0.05, comparing BCP and BCT vs. control.

A pilot 3-day study revealed considerable lung damage in BLEO animals compared to controls, as evidenced by picro-sirius red and H&E staining (p ≤ 0.05, Figures 1A–D). In addition, BLEO instilled animals showed significant increase in RVSP (Figure 1E). Based on these histological and hemodynamic assessments, we decided to initiate C21 treatment after 3 days of BLEO insult in the treatment protocol (BCT group, *n* = 8). On the other hand, C21 was injected immediately after BLEO instillation in the prevention (BCP) protocol. It is evident from Figures 2A,B that BLEO lungs are stained intense red (represents lung collagen accumulation) as compared to controls, and that this pattern is significantly attenuated by C21 in both BCP and BCT groups. Quantification of the red color collagen staining is provided in Figure 2D. Likewise, hydroxyproline analysis revealed that C21 treatment normalized BLEO-induced increase in hydroxyproline levels in both BCP and BCT groups (*p* ≤ 0.05, Figure 2E). Further, Ashcroft scoring of the H&E stained lung sections showed considerable disruption of the lung architecture (especially collapsed alveoli) upon bleomycin instillation, which was attenuated by C21 administration (*p* ≤ 0.05, Figures 2C,F).

### C21 treatment improves ventricular remodeling and hemodynamics in bleomycin animals

**Figure 3:**
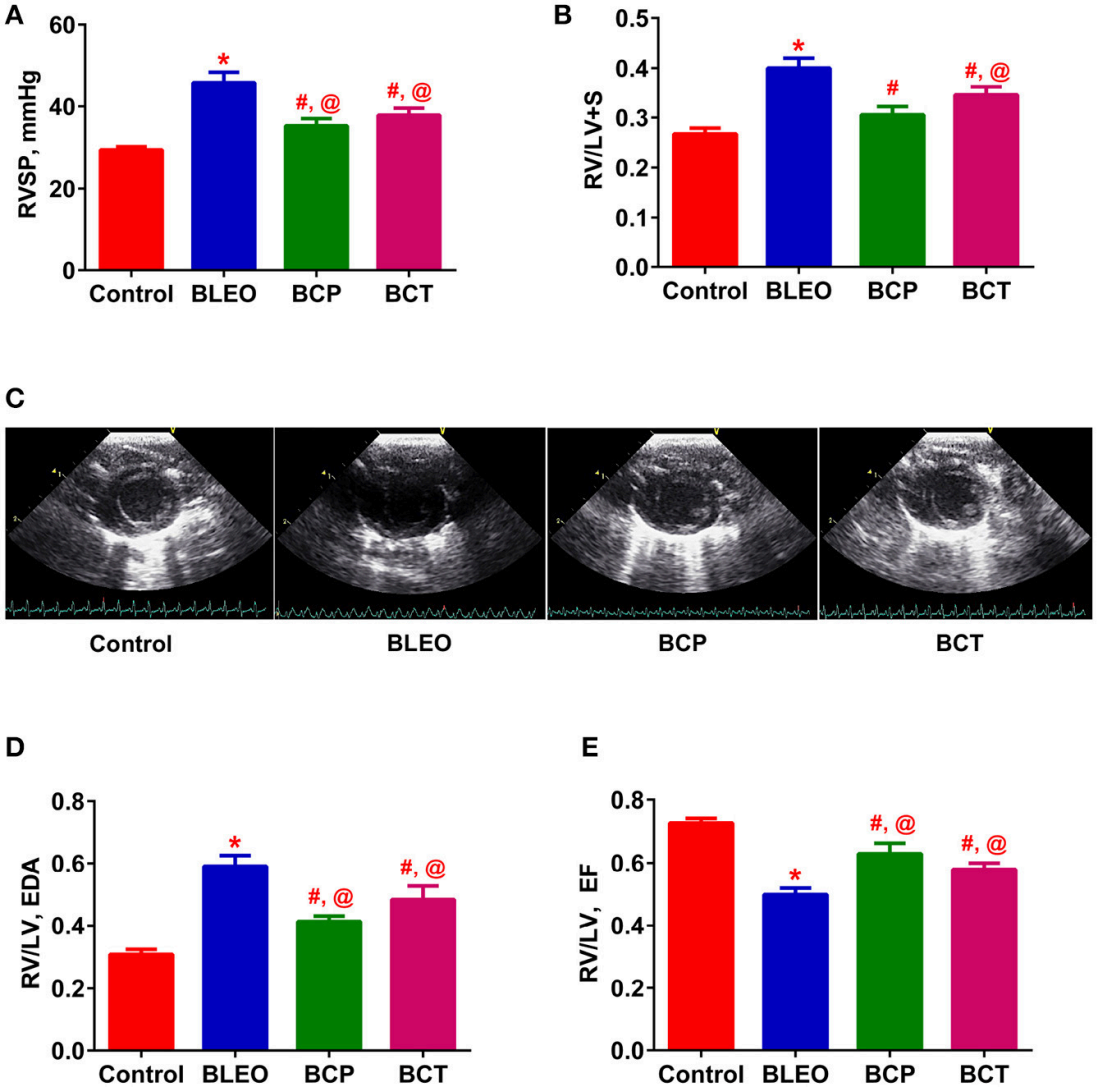
C21 treatment attenuates the right ventricular remodeling in BLEO animals. **(A,B,D,E)** Summary data showing the improvement of RVSP, RVH, RV/LV EDA, and RV/LV EF in BLEO animals in presence of C21. **(C)** Representative images of parasternal short axis view of the ventricles on Day-14. Data represented in **(A,B,D,E)** are mean ± SEM (*n* = 6–8). ^*^Indicates a *p*-value of ≤ 0.05, comparing BLEO animals against the controls. # Indicates a *p*-value of ≤ 0.05, comparing BCP and BCT vs. BLEO. @ Indicates a *p*-value of ≤ 0.05, comparing BCP and BCT vs. control.

Following 2 weeks of BLEO instillation, RVSP was significantly increased in the BLEO group as compared to controls (*p* ≤ 0.05, Figure 3A). This increase in pressure was associated with development of RVH (*p* ≤ 0.05, Figure 3B). However, administration of C21 substantially attenuated the altered hemodynamics and RVH in both the experimental protocols (*p* ≤ 0.05, Figures 3A,B). Further, analysis of the ECHO images revealed a shift in the intra-ventricular septum toward the left ventricle in BLEO-instilled animals, which could be due to elevated RVSP. An increase in RV/LV end diastolic area (EDA) was also observed in BLEO animals that was accompanied with decreased ejection fraction (EF) (*p* ≤ 0.05, Figures 3C,E). In line with the hemodynamics and RVH data, C21 treatment significantly improved the cardiac function in both the experimental protocols (*p* ≤ 0.05, Figures 3D,E). Although C21 treatment was beneficial in improving hemodynamic and all cardiac parameters in the prevention protocol, statistical analysis revealed that only RVH was completely prevented (BCP group), while all other parameters showed partial attenuation in both the experimental groups.

### C21 treatment improves pulmonary vascular remodeling in bleomycin animals

**Figure 4:**
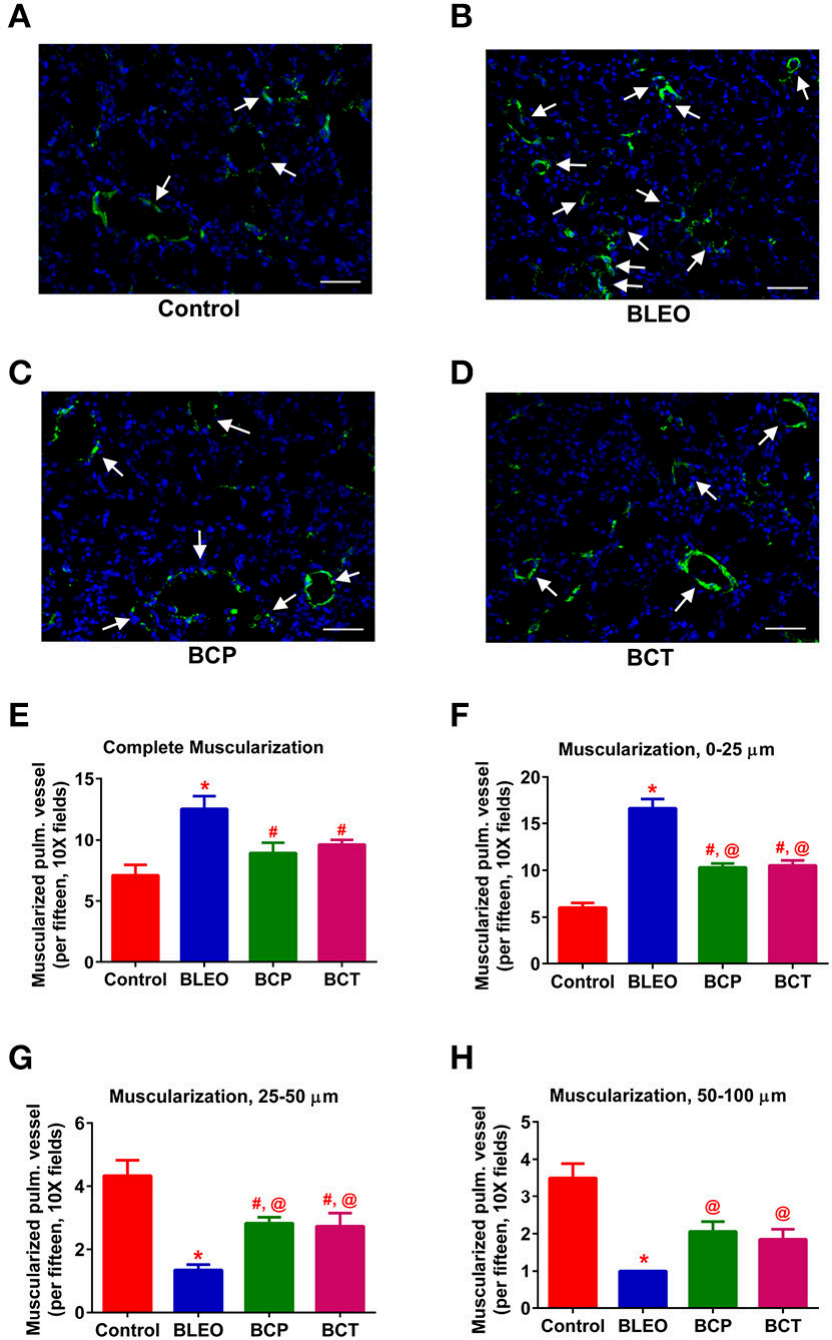
C21 treatment attenuates pulmonary vascular remodeling in BLEO animals. **(A–D)** Representative images of α-smooth muscle actin stained pulmonary vessel wall (white arrows). **(E–H)** Summary data showing the improvement of muscularization by C21 in BLEO animals for the complete, 0–25, 25–50, and 50–100 μm category, respectively. Data represented in **(E–H)** are mean ± SEM (*n* = 3). ^*^Indicates a *p*-value of ≤ 0.05, comparing BLEO animals against the controls. # Indicates a *p*-value of ≤ 0.05, comparing BCP and BCT vs. BLEO. @ indicates a *p*-value of ≤ 0.05, comparing BCP and BCT vs. control. Scale = 50 μm.

In order to investigate remodeling of the pulmonary vasculature, we performed α-smooth muscle actin staining and quantified the degree of muscularization. We observed extensive remodeling of the BLEO lungs as evidenced by an increase in the number of completely muscularized pulmonary vessels (0–100 μm, *p* ≤ 0.05, Figures 4A–E), as well as muscularization of the smaller vessels (*p* ≤ 0.05, Figures 4F–H). In agreement with the hemodynamics and cardiac function data, C21 treatment significantly reduced muscularization of the pulmonary vessels in both the experimental groups, when compared against the BLEO group (*p* ≤ 0.05, Figures 4E–G). However, a detailed statistical analysis revealed that there was total prevention of the completely muscularized vessels, while other muscularization outcomes were partially prevented in the prevention protocol. Likewise, though statistically significant only partial attenuation of muscularization was observed in the BCT group.

### C21 treatment attenuates the expression of ECM and inflammatory markers, along with decreasing macrophage infiltration in bleomycin animals

**Figure 5:**
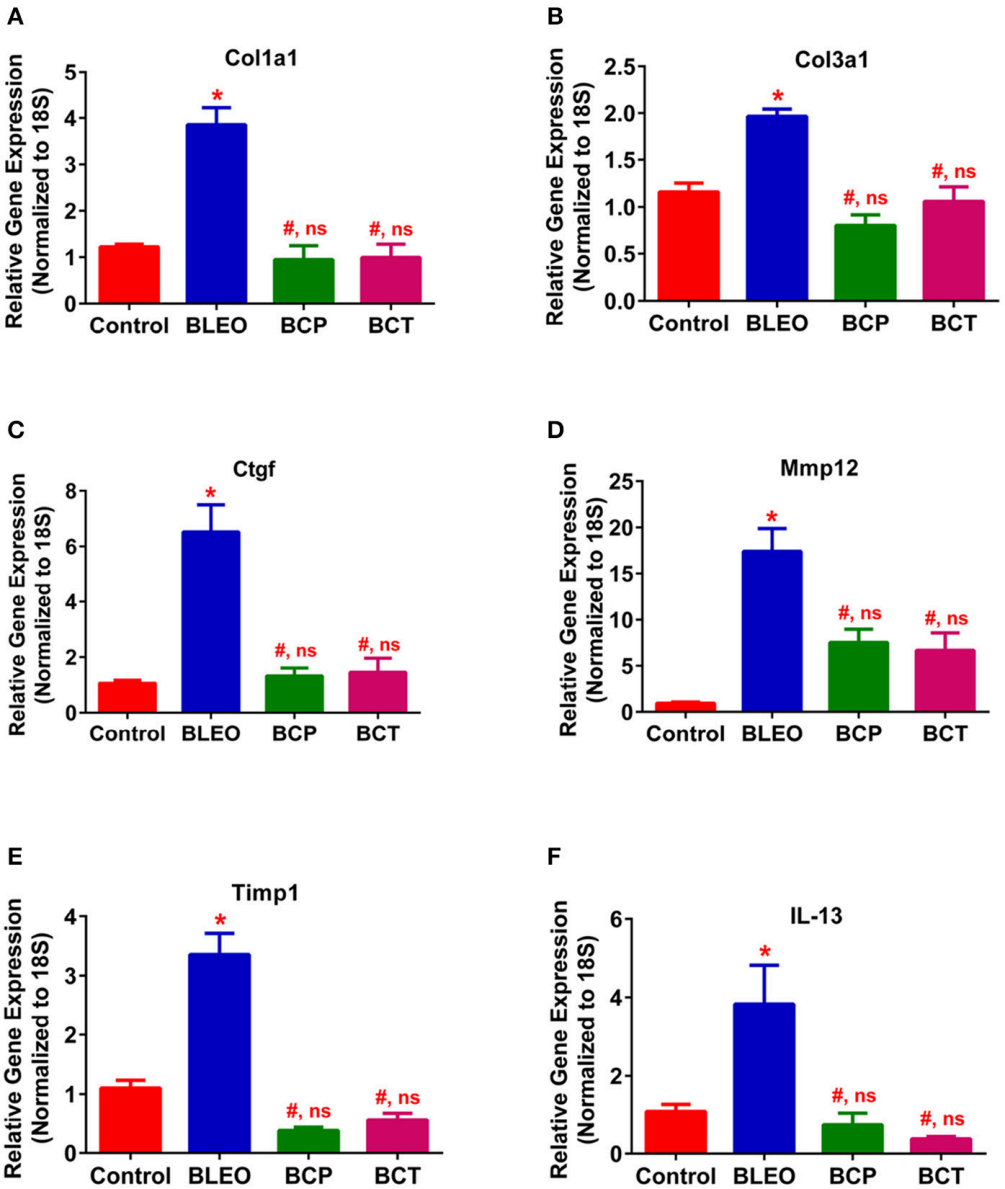
C21 treatment attenuates the expression of markers of fibrosis and ECM in BLEO animals. **(A)** Col1a1, **(B)** Col3a1, **(C)** Ctgf, **(D)** Mmp12, **(E)** Timp1, and **(F)** IL-13. Data represented in **(A–F)** are mean ± SEM, *n* = 5 animals/group. ^*^Indicates a *p*-value of ≤ 0.05, comparing BLEO animals against the controls. # Indicates a *p*-value of ≤ 0.05, comparing BCP and BCT vs. BLEO. ns indicates a *p*-value of non-significant, comparing BCP and BCT vs. control.

**Figure 6:**
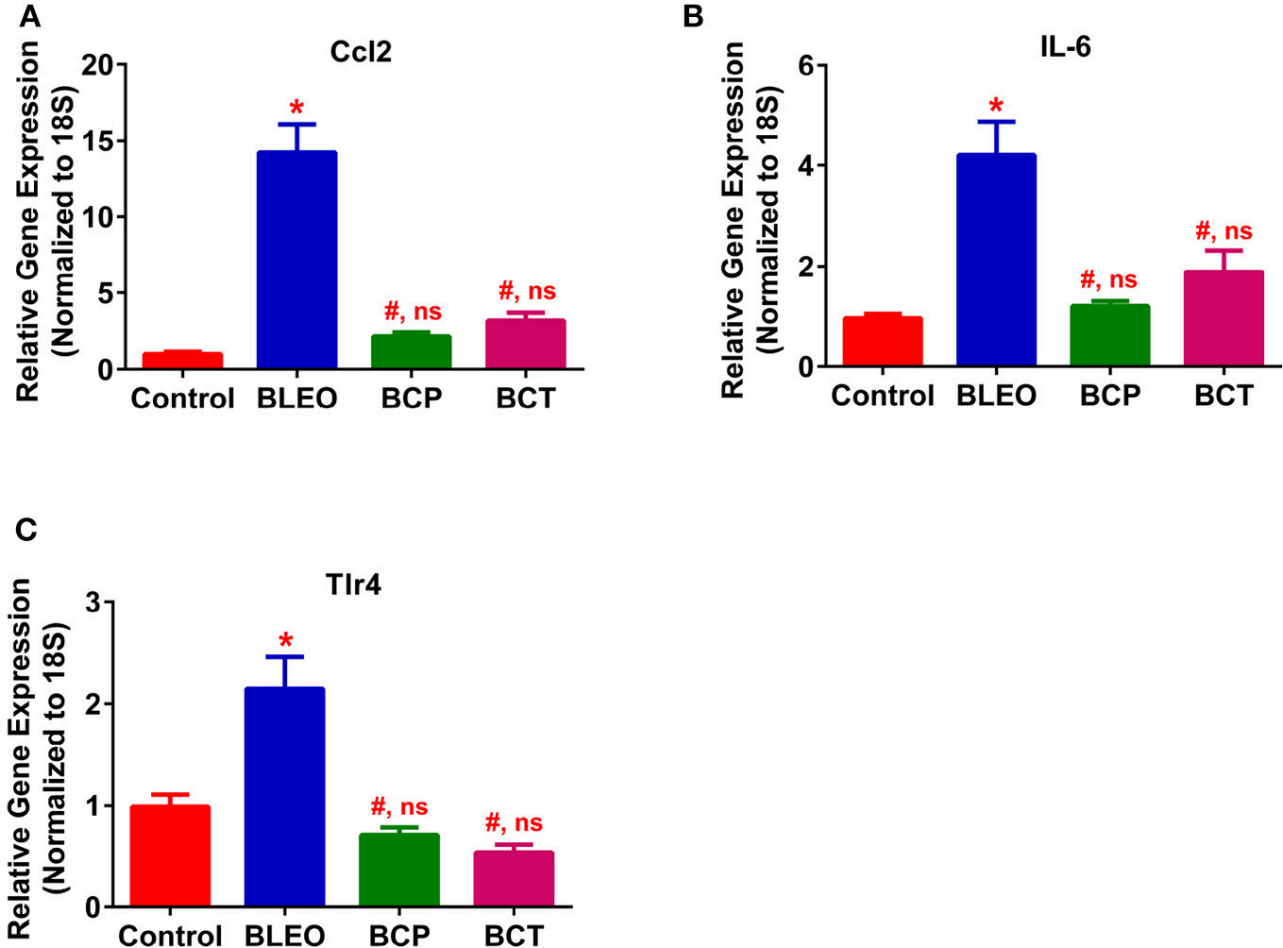
C21 treatment attenuates the expression of inflammatory markers in BLEO animals **(A)** Ccl2, **(B)** IL-6, and **(C)** Tlr4. Data represented in **(A–C)** are mean ± SEM, *n* = 5 animals/group. ^*^Indicates a *p*-value of ≤ 0.05, comparing BLEO animals against the controls. # Indicates a *p*-value of ≤ 0.05, comparing BCP and BCT vs. BLEO. ns indicates a *p*-value of non-significant, comparing BCP and BCT vs. control.

**Figure 7:**
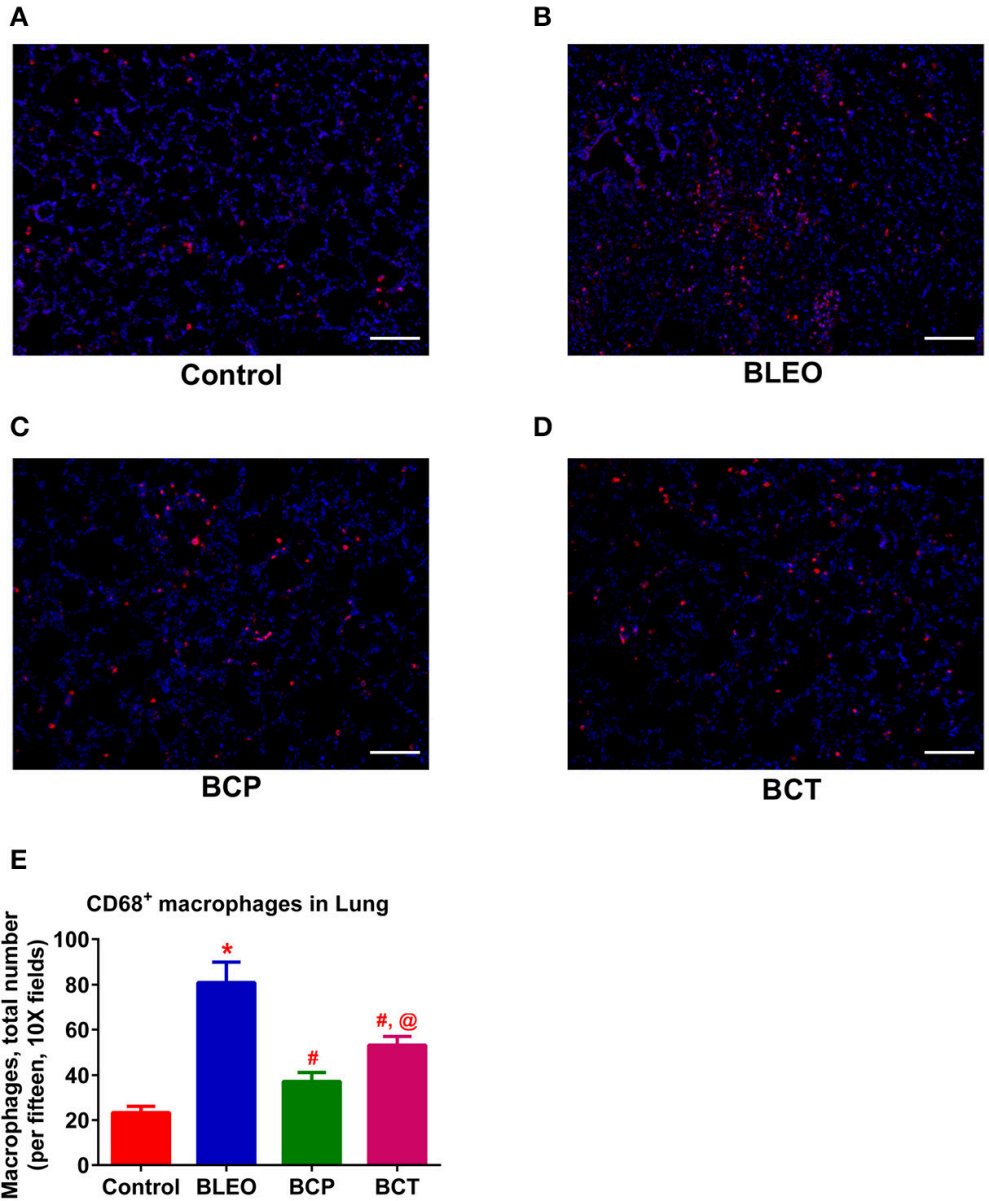
C21 treatment reduces the infiltration of pulmonary macrophages in BLEO animals **(A–D)** Representative images of CD68^+^ macrophages. **(E)** Summary data showing the reduction of CD68^+^ pulmonary macrophages (red) in BLEO animals in presence of C21. Data represented in **(E)** is mean ± SEM (*n* = 3). ^*^Indicates a *p*-value of ≤ 0.05, comparing BLEO animals against the controls. # Indicates a *p*-value of ≤ 0.05, comparing BCP and BCT vs. BLEO. @ indicates a *p*-value of ≤ 0.05, comparing BCP and BCT vs. control. Scale = 100 μm.

To investigate the possible mechanism (or pathways) by which C21 attenuates lung fibrosis, real time qRT-PCR determinations and immunohistochemical staining of the lung macrophages were performed. Gene expression of Col1a1 and Col3a1 was found to be significantly upregulated in BLEO animals, but was attenuated by C21 treatment (*p* ≤ 0.05, Figures 5A,B). Similarly, C21 treatment significantly attenuated the gene expression of Ctgf, Mmp12, Timp1 and Il-13) in both the experimental protocols (*p* ≤ 0.05, Figures 5C–F). Further, the key markers of inflammation, (Ccl2 and Il-6) and innate immune system (Tlr4) were also significantly upregulated in the BLEO animals, but significantly attenuated by C21 (*p* ≤ 0.05, Figures 6A–C). As a next step, we performed immunohistochemical staining to estimate the infiltration of macrophages in the lungs. In line with our gene expression results, BLEO lungs exhibited significant infiltration of CD68^+^ macrophages, which was attenuated by C21 treatment in both the experimental groups (*p* ≤ 0.05, Figures 7A–E). However, statistical analysis revealed that macrophage infiltration was normalized only in the BCP group, while it was partially attenuated in the BCT group.

### C21 treatment alters the expression of angiotensin type 2 receptor in bleomycin animals

**Figure 8:**
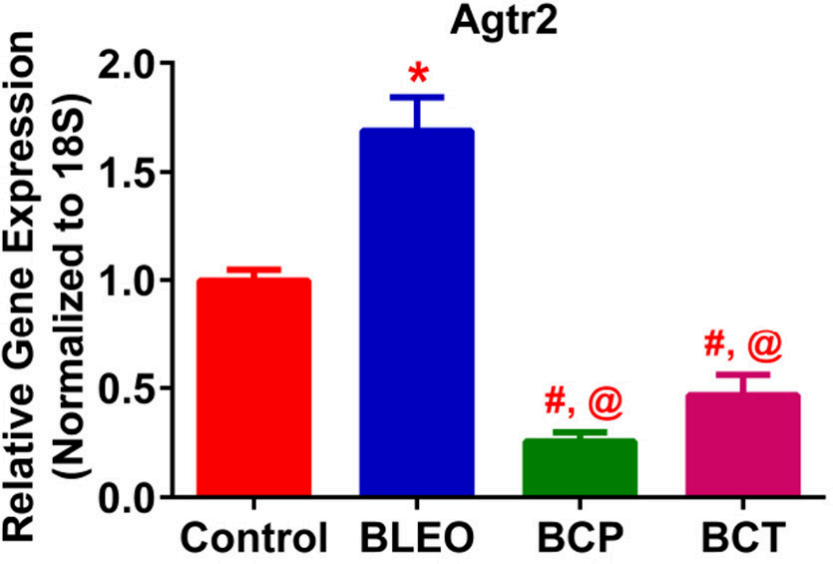
C21 treatment attenuates the expression of Angiotensin type 2 receptor in BLEO animals. Treatment of BLEO animals with C21 attenuated the expression of Agtr2 in the lungs. Data represented is mean ± SEM, *n* = 5 animals/group. ^*^Indicates a *p*-value of ≤ 0.05, comparing BLEO animals against the controls. #Indicates a *p*-value of ≤ 0.05, comparing BCP and BCT vs. BLEO. @ indicates a *p*-value of ≤ 0.05, comparing BCP and BCT vs. control.

qRT-PCR determinations revealed that the expression of Agtr2 was considerably upregulated in the BLEO animals as compared with controls (*p* ≤ 0.05, Figure 8). However, C21 treatment significantly reduced the expression of Agtr2 in both the experimental groups (*p* ≤ 0.05, Figure 8).

## Discussion

The most significant finding of the present work is that pharmacological activation of the AT_2_ receptor by C21 effectively mitigates PF, and improves cardiopulmonary complications in an experimental model of bleomycin-induced lung injury. We observed that C21 treatment of BLEO animals significantly: (1) reduces PH and decreases muscularization of the pulmonary vessels, along with restoring cardiac function; (2) prevents as well as attenuates the progression of PF by reducing ECM remodeling and lung collagen accumulation, and; (3) alleviates inflammatory stress by reducing the infiltration of lung macrophages. Collectively, these data suggest a potential role of the AT_2_ receptor and its synthetic activator (C21) in the treatment of patients with PF and associated Group III PH.

PH due to interstitial lung disease (IPF and chronic obstructive pulmonary disease) has been classified as Group III PH by the WHO. There is an increased incidence of PH and right heart failure in patients with IPF, which adversely affects disease outcomes and survival. To our knowledge, this is the first report to show that stimulation of the AT_2_ receptor using a synthetic activator (C21) renders protection against bleomycin-induced cardiopulmonary injury, which could open up novel therapeutic avenues to tackle lung fibrosis and Group III PH. We chose to evaluate the effects of C21 in the BLEO-model since it mimics key pathological features of human IPF and associated PH. We have previously demonstrated that a single intra-tracheal injection of BLEO induces fibrosis and alters pulmonary hemodynamics within 14 days of insult (Shenoy et al., 2013; Rathinasabapathy et al., 2016). In our experience, remodeling of the lung tissue and changes in pulmonary hemodynamics commence as early as 3 days, which worsens over time, causing considerable mortality after 15 days of intra-tracheal BLEO injection. For this reason, we terminate our experiments after 2-weeks of BLEO instillation. It was interesting to observe that markers of fibrosis and pulmonary pressure were elevated just after 3 days of bleomycin instillation. Thus, it appears that the pathology of PF and PH occur simultaneously in the bleomycin model. Though it is logical to believe that PH follows fibrotic lung injury, it is possible that both the pathological conditions might occur concurrently. From our study, it is difficult to figure out which pathology comes first—PF or PH. Additional studies in the bleomycin model are warranted to evaluate and clarify these points.

Right ventricular structure and function is often altered in patients with IPF because of increased pulmonary vascular resistance and cardiac overload (Han et al., 2013; Rivera-Lebron et al., 2013). In our animal study, bleomycin-instillation was associated with ventricular hypertrophy and right heart dysfunction. Interestingly, treatment with C21 not only attenuated bleomycin-induced cardiac hypertrophy but also improved right heart function in both the experimental protocols. It is conceivable that the protective effects of C21 on the heart may be secondary to a reduction in fibrotic lung injury. However, it is also possible that C21 may exhibit direct actions on the heart. In fact, C21 has been shown to exert protective actions against experimental models of cardiovascular injury (Kaschina et al., 2008; Rehman et al., 2012). With regards to pirfenidone and nintedanib (the approved anti-fibrotic drugs), there exists limited data in the literature on the cardioprotective effects of these agents in the bleomycin model. However, pirfenidone, has been shown to produce beneficial actions on the heart (Avila et al., 2014). It would be interesting to evaluate the cardiopulmonary effects of a combination therapy with C21 and pirfenidone/nintedanib. In conjunction to improving the heart function, C21 treatment also improved pulmonary vascular remodeling by reducing the muscularization of smaller vessels, as evidenced by histology. A potential limitation of our study is that the lungs of BLEO-injected animals at the onset of C21 administration in the treatment protocol (Day 4) resembles early stage of IPF, and not an advanced disease stage. Further studies are warranted to evaluate the effects of C21 on the advanced stage of lung fibrosis by commencing C21 treatment after 7 or 10 days of BLEO instillation. One another limitation is that we have not conducted antagonist experiments with an AT_2_ receptor blocker (PD123319) to convincingly show that C21 mediates its anti-fibrotic effects via stimulation of the AT_2_ receptor. Additional studies need to be performed with an AT_2_ receptor blocker to address this deficiency. However, at this point we contend that the significant findings observed in the present investigation overrules the above mentioned potential limitations and ascertains that C21 could indeed be an effective strategy for the treatment of IPF and Group III PH.

Accumulating evidence indicate that the RAS has important functions within the cardiopulmonary system. While the role of the AT_1_ receptor has been well-recognized, little is known about the functional significance of the AT_2_ receptor. The reason being, there is a relatively lower expression of AT_2_ receptor in the adult tissue. However, during injury, the receptor expression levels increase significantly (Matavelli and Siragy, 2015). There have been conflicting reports on whether the observed increase in AT_2_ receptor during injury contributes to tissue damage, or that it plays a compensatory protective role. With regards to fibrotic lung injury, Konigshoff et al., reported an upregulation of the AT_2_ receptor in the lungs of IPF patients (Königshoff et al., 2007). Similarly, animal models of lung fibrosis were associated with increased pulmonary AT_2_ receptor levels (Königshoff et al., 2007; Rey-Parra et al., 2012). Consistent with these reports, we also observed an elevation in the expression of lung AT_2_ receptor in BLEO animals.

AT_2_ receptor is primarily expressed by lung epithelial cells (Bullock et al., 2001), fibroblasts, and activated myofibroblasts (Königshoff et al., 2007). We propose that the observed upregulation of AT_2_ receptor in BLEO animals could arise from increased numbers of lung fibroblast/myofibroblast, a common feature of fibrotic lung injury (Pardo and Selman, 2016). However, the AT_2_ receptor levels were significantly lower in C21 treated animals, which correlated with reduced pathology. Further support that the AT_2_ receptors are found on the myofibroblasts comes from a recent report by Kumar et al. (2016) who demonstrated that in cultured human lung fibroblasts (MRC5 cells), BLEO (5 μg/ml) triggered the differentiation of fibroblasts into myofibroblasts with a marked elevation of expression level of fibrotic markers, fibronectin, alpha smooth muscle actin, collagen type1, and collagen type 3. Co-treatment of these cells with C21 (10 μg/ml) blocked the expression of these markers (Kumar et al., 2016). Thus, a plausible explanation for our *in vivo* findings is that C21 may inhibit fibroblast proliferation or differentiation, and/or reduce the influx of circulating fibrocytes. Since there is less fibroblast/myofibroblast with C21 treatment, there is less AT_2_ receptor in the treated group. On the contrary, in the monocrotaline-induced PH model, we have reported that the AT_2_ levels increase with C21 treatment. These contrasting findings could be attributed to differences in disease models (monocrotaline vs. bleomycin), the time of C21 treatment initiation, and/or the different lung cell-types involved in the disease pathology. We believe that identification of cell-types that express the AT_2_ receptor in these two varied models of lung injury (Bleomycin and Monocrotaline) would be beneficial and help to resolve some of the observed discrepancy.

Lung fibroblasts are actively involved in the secretion of collagen type I, type III, and fibronectin, thus contributing to the accumulation of ECM proteins and fibrogenesis (Shimbori et al., 2013). The synthesis rates of types I and III collagen are regulated by the mRNA expression levels of Col1a1 and Col3a1, respectively. We observed that the gene expression of Col1a1 and Col3a1 are significantly upregulated in BLEO lungs, but normalized with C21 treatment in both the experimental protocols. Our results are in agreement with the recent findings, which demonstrated that stimulation of the AT_2_ receptor with C21 suppresses collagen synthesis to exhibit anti-fibrotic actions in diverse models of organ fibrosis (Castoldi et al., 2016; Chow et al., 2016). Apart from collagen, a wide variety of matrix metalloproteinases (MMPs) and endogenous inhibitors of metalloproteinases (TIMPs, tissue inhibitor of MMPs) are involved in lung fibrogenesis (Giannandrea and Parks, 2014). In addition, upregulation of Il-13 and Ctgf (Guo et al., 2015; Yang et al., 2015) expression levels contribute to fibrotic lung diseases. Hence, we wanted to investigate the effects of C21 on the gene expression of Mmp 12, Timp1, Il-13, and Ctgf. Interestingly, stimulation of the AT_2_ receptor by C21 decreased levels of Mmp12, Timp1, Il-13, and Ctgf in both the experimental protocols. Our results are in line with the published literature (Koulis et al., 2015). Thus, the anti-fibrotic effects of C21 may be partly mediated by modulation of MMP's, TIMP's, Il-13, and CTGF.

Increasing evidence suggests that pharmacological activation of the AT_2_ receptor attenuates inflammation and oxidative stress to exert anti-fibrotic actions (Koulis et al., 2015). In the present study, treatment of BLEO animals with C21 resulted in significant downregulation of Ccl2, Il-6, and Tlr4 in combination with reduced recruitment or infiltration of macrophages to the injured lung, could contribute to the observed protective effects. A recent review on AT_2_ receptor agonists summarizes that the receptor activation by C21 mechanistically arrests and attenuates the multiple process of fibrosis by: (i) the direct inhibition of pro-inflammatory and fibrotic factors; (ii) inhibition of remodeling of macrophages and fibroblasts to myofibroblasts; and (iii) inhibition of secretion or synthesis of ECM or collagen by myofibroblasts (Wang Y. et al., 2017). Overall, these findings strongly support our contention that the activation of the AT_2_ receptor by C21 has a direct role in providing anti-inflammatory and anti-fibrotic actions.

**Figure 9:**
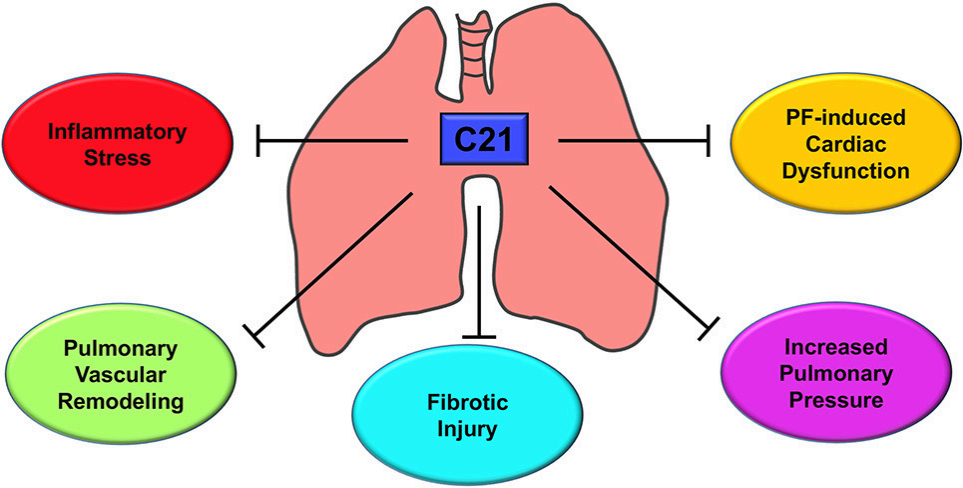
Putative mechanism of action - AT_2_R stimulation by C21. C21 exerts a host of actions on the cardiopulmonary system that include decrease in lung inflammatory stress, reduction of pulmonary vascular remodeling, attenuation of fibrotic lung injury, lowering of pulmonary pressure, and improvement of cardiac function. All these properties are responsible for the protective actions of C21 against pulmonary fibrosis and associated cardiac damage.

To improve life expectancy in patients with Group III PH due to fibrotic lung injury, it is preemptive to treat cardiovascular complications since fibrosis is an irreversible process. In this investigation, we have demonstrated that activation of the AT_2_ receptor by C21 significantly decreased inflammatory stress, reduced pulmonary vascular remodeling, mitigated fibrotic lung injury, restored pulmonary hemodynamics, and attenuated cardiac dysfunction as represented in Figure 9. Collectively, our study provides the necessary experimental evidence to attempt the strategy of utilizing AT_2_ receptor agonist for the treatment of IPF and Group III PH.

## Author contributions

AR, MK, MR, and VS: Conceived and designed the experiment; AR, AH, KH, AK, DM, SG, GB, and VS: Performed the experiment; AR, TU, MR, JW, US, CS, MK, and VS: Analyzed the data; AR, US, CS, MK, and VS: Wrote the paper.

### Conflict of interest statement

The authors declare that the research was conducted in the absence of any commercial or financial relationships that could be construed as a potential conflict of interest.

## Abbreviations

Agtr2: angiotensin type 2 receptor
Ang-(1-7): Angiotensin-(1-7)
AT_1_: angiotensin type 1
AT_2_: angiotensin type 2
BLEO: bleomycin
BCP: bleomycin + compound 21 (prevention)
BCT: bleomycin + compound 21 (treatment)
Col1a1: Collagen, type 1, a1
Col3a1: Collagen, type3, a1
Ctgf: Connective Tissue Growth Factor
C21: compound 21
ECHO: echocardiography
ECM: extracellular matrix
EDA: end diastolic area
EF: ejection fraction
Il-6: Interleukin 6
Il-13: Interleukin 13
IPF: Idiopathic Pulmonary Fibrosis
LV: left ventricle
MMP: matrix metalloproteinases
MCP-1: monocyte chemoattractant protein 1
PF: pulmonary fibrosis
PH: Pulmonary Hypertension
RAS: renin angiotensin system
RVH: right ventricular hypertrophy
RVSP: right ventricular systolic pressure
TIMP: tissue inhibitor of metalloproteinase
TLR4: Toll Like Receptor 4.

## References

[B1] Anandharajan R. Sayyed S. G. Doshi L. S. Dixit P. Chandak P. G. Dixit A. V.. (2009). 18F9 (4-(3,6-bis (ethoxycarbonyl)-4,5,6,7-tetrahydrothieno (2,3-c) pyridin-2-ylamino)-4-oxobutanoic acid) enhances insulin-mediated glucose uptake *in vitro* and exhibits antidiabetic activity *in vivo* in db/db mice. Metab. Clin. Exp. 58, 1503–1516. 10.1016/j.metabol.2009.04.036 19608207

[B2] Ashcroft T. Simpson J. M. Timbrell V. (1988). Simple method of estimating severity of pulmonary fibrosis on a numerical scale. J. Clin. Pathol. 41, 467–470. 10.1136/jcp.41.4.467 3366935 PMC1141479

[B3] Avila G. Osornio-Garduño D. S. Ríos-Pérez E. B. Ramos-Mondragón R. (2014). Functional and structural impact of pirfenidone on the alterations of cardiac disease and diabetes mellitus. Cell Calcium 56, 428–435. 10.1016/j.ceca.2014.07.008 25108569

[B4] Bruce E. Shenoy V. Rathinasabapathy A. Espejo A. Horowitz A. Oswalt A.. (2015). Selective activation of angiotensin AT2 receptors attenuates progression of pulmonary hypertension and inhibits cardiopulmonary fibrosis. Br. J. Pharmacol. 172, 2219–2231. 10.1111/bph.13044 25522140 PMC4403089

[B5] Bullock G. R. Steyaert I. Bilbe G. Carey R. M. Kips J. De Paepe B.. (2001). Distribution of type-1 and type-2 angiotensin receptors in the normal human lung and in lungs from patients with chronic obstructive pulmonary disease. Histochem. Cell Biol. 115, 117–124. 10.1007/s004180000235 11444146

[B6] Canestaro W. J. Forrester S. H. Raghu G. Ho L. Devine B. E. (2016). Drug Treatment of Idiopathic Pulmonary Fibrosis: Systematic Review and Network Meta-Analysis. Chest 149, 756–766. 10.1016/j.chest.2015.11.013 26836914

[B7] Castoldi G. di Gioia C. R. Carletti R. Roma F. Zerbini G. Stella A. (2016). Angiotensin Type-2 (AT-2)-Receptor activation reduces renal fibrosis in cyclosporine nephropathy: evidence for blood-pressure independent effect. Biosci. Rep. 36, e00403 10.1042/BSR20160278 PMC5293591 27679859

[B8] Chow B. S. Koulis C. Krishnaswamy P. Steckelings U. M. Unger T. Cooper M. E.. (2016). The angiotensin II type 2 receptor agonist compound 21 is protective in experimental diabetes-associated atherosclerosis. Diabetologia 59, 1778–1790. 10.1007/s00125-016-3977-5 27168137

[B9] Collum S. D. Amione-Guerra J. Cruz-Solbes A. S. DiFrancesco A. Hernandez A. M. Hanmandlu A.. (2017). Pulmonary Hypertension Associated with Idiopathic Pulmonary Fibrosis: Current and Future Perspectives. Can. Respir. J. 2017:1430350. 10.1155/2017/1430350 28286407 PMC5327768

[B10] Giannandrea M. Parks W. C. (2014). Diverse functions of matrix metalloproteinases during fibrosis. Dis. Model. Mech. 7, 193–203. 10.1242/dmm.012062 24713275 PMC3917240

[B11] Guo J. Yao H. Lin X. Xu H. Dean D. Zhu Z.. (2015). IL-13 induces YY1 through the AKT pathway in lung fibroblasts. PLoS ONE 10:e0119039. 10.1371/journal.pone.0119039 25775215 PMC4361578

[B12] Han M. K. Bach D. S. Hagan P. G. Yow E. Flaherty K. R. Toews G. B.. (2013). Sildenafil preserves exercise capacity in patients with idiopathic pulmonary fibrosis and right-sided ventricular dysfunction. Chest 143, 1699–1708. 10.1378/chest.12-1594 23732584 PMC3673665

[B13] Iwai M. Horiuchi M. (2009). Devil and angel in the renin-angiotensin system: ACE-angiotensin II-AT1 receptor axis vs. ACE2-angiotensin-(1-7)-Mas receptor axis. Hypertens Res. 32, 533–536. 10.1038/hr.2009.74 19461648 PMC7091931

[B14] Joseph J. P. Mecca A. P. Regenhardt R. W. Bennion D. M. Rodríguez V. Desland F.. (2014). The angiotensin type 2 receptor agonist Compound 21 elicits cerebroprotection in endothelin-1 induced ischemic stroke. Neuropharmacology 81, 134–141. 10.1016/j.neuropharm.2014.01.044 24508710 PMC7472595

[B15] Kaschina E. Grzesiak A. Li J. Foryst-Ludwig A. Timm M. Rompe F.. (2008). Angiotensin II type 2 receptor stimulation: a novel option of therapeutic interference with the renin-angiotensin system in myocardial infarction? Circulation 118, 2523–2532. 10.1161/CIRCULATIONAHA.108.784868 19029468

[B16] Königshoff M. Wilhelm A. Jahn A. Sedding D. Amarie O. V. Eul B.. (2007). The angiotensin II receptor 2 is expressed and mediates angiotensin II signaling in lung fibrosis. Am. J. Respir. Cell Mol. Biol. 37, 640–650. 10.1165/rcmb.2006-0379TR 17630322

[B17] Koulis C. Chow B. S. McKelvey M. Steckelings U. M. Unger T. Thallas-Bonke V.. (2015). AT2R agonist, compound 21, is reno-protective against type 1 diabetic nephropathy. Hypertension 65, 1073–1081. 10.1161/HYPERTENSIONAHA.115.05204 25776077

[B18] Kumar A. Rathinasabapathy A. Horowitz A. Horton K. Martinez D. Raizada M. (2016). Stimulation of AT2 receptor decreases collagen expression and inhibits myofibroblast differentiation to protect against bleomycin-induced pulmonary fibrosis. FASEB J. 30(1 Suppl), lb586 10.1096/fasebj.30.1_supplement.lb586

[B19] Ley B. Collard H. R. (2013). Epidemiology of idiopathic pulmonary fibrosis. Clin. Epidemiol. 5, 483–492. 10.2147/CLEP.S54815 24348069 PMC3848422

[B20] Matavelli L. C. Siragy H. M. (2015). AT2 receptor activities and pathophysiological implications. J. Cardiovasc. Pharmacol. 65, 226–232. 10.1097/FJC.0000000000000208 25636068 PMC4355033

[B21] Nogueira-Ferreira R. Faria-Costasup G. Ferreira R. Henriques-Coelho T. (2016). Animal models for the study of pulmonary hypertension: potential and limitations. Cardiol. Cardiovasc. Med. 1, 1–22. 10.26502/fccm.9292001

[B22] Pardo A. Selman M. (2016). Lung fibroblasts, aging, and idiopathic pulmonary fibrosis. Ann. Am. Thorac Soc. 13(Suppl. 5), S417–S421. 10.1513/AnnalsATS.201605-341AW 28005427

[B23] Patel S. N. Ali Q. Hussain T. (2016). Angiotensin II type 2-receptor agonist C21 reduces proteinuria and oxidative stress in kidney of high-salt-fed obese zucker rats. Hypertension 67, 906–915. 10.1161/HYPERTENSIONAHA.115.06881 27021008 PMC4833537

[B24] Raghu G. Collard H. R. Egan J. J. Martinez F. J. Behr J. Brown K. K.. (2011). An official ATS/ERS/JRS/ALAT statement: idiopathic pulmonary fibrosis: evidence-based guidelines for diagnosis and management. Am. J. Respir. Crit. Care Med. 183, 788–824. 10.1164/rccm.2009-040GL 21471066 PMC5450933

[B25] Raghu G. Selman M. (2015). Nintedanib and pirfenidone. New antifibrotic treatments indicated for idiopathic pulmonary fibrosis offer hopes and raises questions. Am. J. Respir. Crit. Care Med. 191, 252–254. 10.1164/rccm.201411-2044ED 25635489

[B26] Rathinasabapathy A. Bruce E. Espejo A. Horowitz A. Sudhan D. R. Nair A.. (2016). Therapeutic potential of adipose stem cell-derived conditioned medium against pulmonary hypertension and lung fibrosis. Br. J. Pharmacol. 173, 2859–2879. 10.1111/bph.13562 27448286 PMC5275771

[B27] Rehman A. Leibowitz A. Yamamoto N. Rautureau Y. Paradis P. Schiffrin E. L. (2012). Angiotensin type 2 receptor agonist compound 21 reduces vascular injury and myocardial fibrosis in stroke-prone spontaneously hypertensive rats. Hypertension 59, 291–299. 10.1161/HYPERTENSIONAHA.111.180158 22184324

[B28] Rey-Parra G. J. Vadivel A. Coltan L. Hall A. Eaton F. Schuster M.. (2012). Angiotensin converting enzyme 2 abrogates bleomycin-induced lung injury. J. Mol. Med. 90, 637–647. 10.1007/s00109-012-0859-2 22246130 PMC7080102

[B29] Rivera-Lebron B. N. Forfia P. R. Kreider M. Lee J. C. Holmes J. H. Kawut S. M. (2013). Echocardiographic and hemodynamic predictors of mortality in idiopathic pulmonary fibrosis. Chest 144, 564–570. 10.1378/chest.12-2298 23450321 PMC3734888

[B30] Shenoy V. Gjymishka A. Jarajapu Y. P. Qi Y. Afzal A. Rigatto K.. (2013). Diminazene attenuates pulmonary hypertension and improves angiogenic progenitor cell functions in experimental models. Am. J. Respir. Crit. Care Med. 187, 648–657. 10.1164/rccm.201205-0880OC 23370913 PMC3733435

[B31] Shenoy V. Kwon K. C. Rathinasabapathy A. Lin S. Jin G. Song C.. (2014). Oral delivery of Angiotensin-converting enzyme 2 and Angiotensin-(1-7) bioencapsulated in plant cells attenuates pulmonary hypertension. Hypertension 64, 1248–1259. 10.1161/HYPERTENSIONAHA.114.03871 25225206 PMC4239698

[B32] Shimbori C. Gauldie J. Kolb M. (2013). Extracellular matrix microenvironment contributes actively to pulmonary fibrosis. Curr. Opin. Pulm. Med. 19, 446–452. 10.1097/MCP.0b013e328363f4de 23872861

[B33] Wan Y. Wallinder C. Plouffe B. Beaudry H. Mahalingam A. K. Wu X.. (2004). Design, synthesis, and biological evaluation of the first selective nonpeptide AT2 receptor agonist. J. Med. Chem. 47, 5995–6008. 10.1021/jm049715t 15537354

[B34] Wang L. Wang Y. Li X. Y. Leung P. S. (2017). Angiotensin II type 2 receptor activation with compound 21 augments islet function and regeneration in streptozotocin-induced neonatal rats and human pancreatic progenitor cells. Pancreas 46, 395–404. 10.1097/MPA.0000000000000754 28099262

[B35] Wang Y. Del Borgo M. Lee H. W. Baraldi D. Hirmiz B. Gaspari T. A.. (2017). Anti-fibrotic potential of AT2 receptor agonists. Front. Pharmacol. 8:564. 10.3389/fphar.2017.00564 28912715 PMC5583590

[B36] Wolters P. J. Collard H. R. Jones K. D. (2014). Pathogenesis of idiopathic pulmonary fibrosis. Annu. Rev. Pathol. 9, 157–179. 10.1146/annurev-pathol-012513-104706 24050627 PMC4116429

[B37] Yang Z. Sun Z. Liu H. Ren Y. Shao D. Zhang W.. (2015). Connective tissue growth factor stimulates the proliferation, migration and differentiation of lung fibroblasts during paraquat-induced pulmonary fibrosis. Mol. Med. Rep. 12, 1091–1097. 10.3892/mmr.2015.3537 25815693 PMC4438944

